# Cancer cell-derived immunoglobulin G activates platelets by binding to platelet FcγRIIa

**DOI:** 10.1038/s41419-019-1367-x

**Published:** 2019-01-28

**Authors:** Shuo Miao, Dan Shu, Ying Zhu, Meng Lu, Qingsong Zhang, Youliang Pei, Ao-Di He, Rong Ma, Bixiang Zhang, Zhang-Yin Ming

**Affiliations:** 1 Department of Pharmacology, School of Basic Medicine, Tongji Medical College of Huazhong University of Science and Technology, Wuhan, China; 2 Department of Urology, Affiliated Hospital of Qingdao University, Qingdao, China; 3 Department of Surgery, Tongji Hospital, Tongji Medical College, Huazhong University of Science and Technology, Wuhan, China; 4 The Key Laboratory for Drug Target Research and Pharmacodynamic Evaluation of Hubei Province, Wuhan, China

## Abstract

Tumor-associated thrombosis is the second leading risk factor for cancer patient death, and platelets activity is abnormal in cancer patients. Discovering the mechanism of platelet activation and providing effective targets for therapy are urgently needed. Cancer cell- derived IgG has been reported to regulate development of tumors. However, studies on the functions of cancer cell-derived IgG are quite limited. Here we investigated the potential role of cancer cell-derived IgG in platelet activation. We detected the expression of CD62P on platelets by flow cytometry and analyzed platelet function by platelets aggregation and ATP release. The content of IgG in cancer cell supernatants was detected by enzyme-linked immune sorbent assay. The distribution of cancer-derived IgG in cancer cells was analyzed by immunofluorescence assay. Western blot was performed to quantify the relative expression of FcγRIIa, syk, PLCγ2. The interaction between cancer cell-derived IgG and platelet FcγRIIa was analyzed by co-immunoprecipitation. The results showed that higher levels of CD62P were observed in cancer patients’ platelets compared with that of healthy volunteers. Cancer cell culture supernatants increased platelet CD62P and PAC-1 expression, sensitive platelet aggregation and ATP release in response to agonists, while blocking FcγRIIa or knocking down IgG reduced the activation of platelets. Coimmunoprecipitation results showed that cancer cell-derived IgG interacted directly with platelet FcγRIIa. In addition, platelet FcγRIIa was highly expressed in liver cancer patients. In summary, cancer cell-derived IgG interacted directly with FcγRIIa and activated platelets; targeting this interaction may be an approach to prevent and treat tumor-associated thrombosis.

## Introduction

The association between platelet and cancer has been recognized for centuries, starting with the identification of Trousseau syndrome in 1865^1^. The interaction between tumor cells and platelets was shown to play a key role in malignant progression, and platelet activation and platelets have been identified as potential new drug targets for cancer therapy^2^. It is known that platelets can regulate tumor growth, tumor angiogenesis, and tumor metastasis^3–5^ by virtue of their vast array of surface receptors^6–9^ and secreted products, such as thromboxane^10^, PDGF^11^, and VEGF^12^. Our studies also showed that platelet-derived TGF-β-mediated KLF6 expression and induced the proliferation of hepatocellular carcinoma (HCC) cells^13^. Additionally, tumor cells can induce platelet activation by releasing metabolites, thrombin^14^, and ADP^15^, which serve as an indirect way to activate platelets.

Mitrugno et al. reported that platelet FcγRIIa can mediate platelet–tumor cell cross-talk and that tumor cells directly induce platelet secretion^16^. FcγRIIa, the low-affinity receptor for the constant fragment (Fc) of immunoglobulin G (IgG), is expressed by neutrophils, monocytes, macrophages, and human platelets. Roles for FcγRIIa have been identified in processes mediating interactions between platelets and immune complexes, specific strains of bacteria^17^, and the innate phase proteins serum amyloid P component and C-reactive protein^18^. However, the cancer cell ligand that stimulates platelet activation by FcγRIIa remains to be elucidated.

Traditionally, it was believed that IgG is produced in B lymphocytes and plasma cells. In recent decades, studies have shown that tumor cells^19,20^ can also express IgG. An increasing number of reports have shown that cancer cell-derived IgG is involved in the progression and survival of cancer cells; cancer cell-derived IgG can enhance the growth and proliferation of cancer cells by inducing the production of low levels of reactive oxygen species in vitro and in vivo^21^. Cancer cell-derived IgG regulates LPS-induced proinflammatory cytokine production by binding to TLR4 and enhancing TLR4 expression^22^. However, no study has shown that B lymphocyte-derived IgG can promote tumor progression. In addition, cancer cell-derived IgG shows many different features and functions compared with IgG from B lymphocytes, such as distinct VHDJH recombinations^23^, different gene expression regulatory mechanisms^24^, and different immunoactivity^25^. In addition, the glycosylation patterns between the two IgGs were also quite different^26,27^.

In this study, we used different cancer cells to investigate the role of cancer cell-derived IgG. We first confirmed that cancer cell-derived IgG could mediate platelet activation and that it interacted with platelet FcγRIIa directly. We also found that the expression of platelet FcγRIIa in HCC patients is higher than that in healthy volunteers. These findings suggest that cancer cell-derived IgG may be an important cause of tumor-associated thrombosis and can serve as a diagnostic biomarker and therapeutic target.

## Materials and methods

### Study subjects

Healthy volunteers without a history of hematological diseases (such as platelet and coagulation disorder) and who did not take any drugs in the preceding 2 weeks were recruited for this study. We collected blood samples from cancer patients hospitalized at Tongji Hospital at the Tongji Medical College of Huazhong University of Science and Technology in Wuhan, China. None of the patients involved in our study used any antithrombotic drugs or received any prior cancer treatment.

### Reagents

Thrombin, ADP, and U46619 were ordered from Sigma (St. Louis, MO, USA). Collagen and CHRONO-LUME reagent were purchased from Corp (Havertown, PA, USA). IV.3 Fab was kindly provided by Prof. Peter Newman (Blood Center, Wisconsin, USA). Anti-syk, anti-PLCγ2, and RP215-HRP antibodies were obtained from Santa Cruz Biotechnology (Santa Cruz, CA, USA), Anti-FcγRIIa antibody was obtained from Abcam (Massachusetts, US). Anti-phospho-PLCγ2 (Tyr1217) and anti-phospho-syk (Tyr525/526) antibodies were obtained from Cell Signaling (Beverly, MA, USA).

### Cell culture and cell supernatant preparation

The human cancer cell lines SMMC.7721 (hepatocellular), LoVo (colorectal), SiHa (cervical), DU145 (prostate), MCF7 (breast), and EJ (bladder) were purchased from the Chinese Center for Type Culture Collection (CCTCC, Wuhan, China). SMMC.7721, LoVo and SiHa were cultured in DMEM (HyClone, US) with 10% FBS (Gibco, NY). DU145, MCF7, and EJ were grown in RPMI 1640 with 10% FBS. The siRNAs against the IgG1 constant region (siRNA1 and siRNA2) and the control siRNA (siCN) (Genechem Corporation, China) were transfected into SMMC.7721, EJ and SiHa cells with Lipofectamine^®^ 3000 siRNA Transfection Reagent (Invitrogen, USA). Cells were further analyzed at 72 h after transfection. siRNA sequences were as follows: siRNA1-: 5′-GGUGGACAAGACAGUUGAG-3′, siRNA2: 5′-AGUGCAAGGUCUCCAACAA-3′, and siCN: 5′-UUCUCCGAACGUGUCACGU-3′. For the preparation of the cell supernatant, tumor cells (2 × 10^5^, 1 mL) were seeded in six-well plates and cultured for 12 h; then, the medium was replaced with 1 mL fresh medium. Supernatants were collected after 24 h, and particulates were removed by centrifuging at 1000×*g* for 10 min.

### Enzyme-linked immunosorbent assay

Cancer cell culture supernatants were collected and analyzed for the levels of IgG (LIANK, China) and C-reactive protein (Neobioscience, China) in the supernatant according to the manufacturers’ instructions.

### Determination of cancer cell-derived IgG isotype

Cancer cell culture supernatants were collected, and the subclasses of cancer cell-derived IgGs were determined using a Quantibody Human Ig Isotype Array (RayBiotech, USA). The experimental procedure was performed according to the manufacturer’s instructions.

### Platelet preparation

Human blood was collected from healthy volunteers and cancer patients who had provided informed consent. Washed platelets^28^ were prepared as described previously. Human blood was drawn from the cubital vein without stasis into siliconized vacutainers containing 1:9 (v/v) 3.8% sodium citrate. Platelet-rich plasma (PRP) was obtained by centrifuging whole uncoagulated blood for 10 min at 150×*g*. Platelets were pelleted by centrifuging the PRP fraction at 800×*g* for 10 min. Platelets were washed with Tyrode’s buffer (137 mM NaCl, 13.8 mM NaHCO_3_, 5.5 mM glucose, 2.5 mM KCl, 20 mM HEPES, and 0.36 mM NaH_2_PO_4_; pH 7.4) containing 1 μM PGE_1_ and 2.5 mM EDTA and finally resuspended in Tyrode’s buffer that did not contain PGE_1_ or EDTA. All of the platelet preparations were conducted at room temperature. The resuspended platelet stood for at least 30 min before the experiment.

### Platelet aggregation and ATP release assay

Platelet aggregation and ATP release assays were performed as described previously^29^. The platelet pellet was resuspended in Tyrode–HEPES buffer, the concentration was adjusted to 900 × 10^9^/L, and the platelets were preincubated at 37 °C with cancer cell supernatant or culture medium (final concentration was 300 × 10^9^/L) for 15 min. CaCl_2_ (1 mM) was added prior to agonist stimulation. Platelet secretion was determined by measuring the release of ATP using a luciferin–luciferase reagent (CHRONO-LUME, Chrono-Log, USA). The quantification was calculated from the actual aggregation and release plots.

### Flow cytometric analysis

Platelets (300 × 10^9^/L, 50 μL) were incubated with cancer cell supernatant or culture medium (100 μL) for 15 min, and the binding of FITC-conjugated anti-CD62P or anti-PAC-1 antibody to human platelets was conducted by incubation in the dark at room temperature for 15 min and analysis with a BD Biosciences flow cytometer (San Jose, USA).

### Western blot analysis

Platelets (900 × 10^9^/L) were preincubated with cancer cell supernatant (1:2, V/V) for 15 min and lysed in 2× lysis buffer on ice. The proteins were separated by 10% SDS–PAGE, transferred to PVDF membranes, blocked with 5% milk in TBST (50 mM Tris (pH 7.5), 250 mM NaCl, and 0.2% Tween 20) and probed with antibodies overnight at 4 °C. The membranes were washed three times with TBST and incubated with the appropriate secondary antibodies for 1 h at room temperature. Immunoreactive bands were visualized using a DNR Bio-imaging system according to the manufacturer’s instructions and quantified by ImageJ software.

### Co-immunoprecipitation (Co-IP)

Cell cultures were digested with trypsin to collect the cancer cells, and then the cell suspensions were carefully washed twice with PBS, resuspended in PBS and sonicated. High-speed centrifugation (13,000 rpm for 15 min) was used to collect the cell supernatant, which was incubated with the platelets for 30 min. Cells were lysed with 2× lysis buffer on ice for 15 min. Cell lysates were centrifuged for 15 min at 13,000 rpm to pellet insoluble material, 2 μg RP215 or irrelevant IgG antibody was added, and the solutions were incubated at 4 °C overnight. Then, 40 μL protein A/G agarose was added, and the solution was incubated with gentle mixing for 2–3 h at 4 °C. Complexes were washed three times in PBS at 4 °C, boiled in 50 μL SDS–PAGE buffer and immunoblotted.

### Statistical analysis

Data were analyzed using GraphPad Prism version 5.0 software. The results are expressed as the mean ± standard error of the mean (SEM). Differences between groups were evaluated using two-tailed Student’s *t*-test or one-way ANOVA. For all statistical analyses, *P* < 0.05 was considered significant.

## Results

### Platelet hyperactivity in cancer patients

**Fig. 1:**
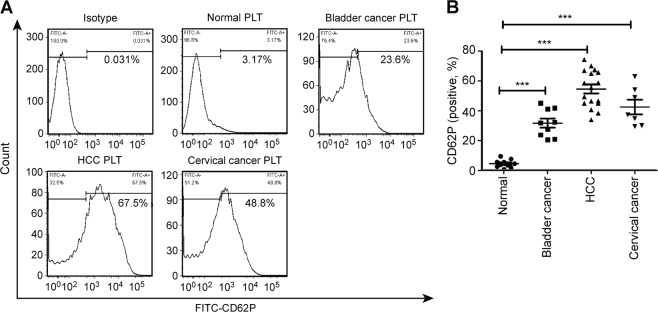
Platelet activity is increased in cancer patients. Washed platelets from healthy volunteers (*n* = 20) and patients with hepatocellular carcinoma (HCC) (*n* = 30), bladder cancer (*n* = 22) or cervical cancer (*n* = 30) were prepared, and the expression of CD62P was detected by flow cytometry. Representative curves are shown in **a**. The corresponding scatter diagram is shown in **b** and the data are represented as the mean ± SEM. ***P < 0.001 compared with normal platelet. PLT platelet

We collected fresh blood samples from healthy volunteers (*n* = 20), HCC patients (*n* = 30), cervical cancer patients (*n* = 30), and bladder cancer patients (*n* = 22). We prepared and washed the platelets and detected the expression of CD62P (also known as p-selectin), which rapidly translocates to the cell surface of platelets upon activation. As the results showed, the rate of positive platelet CD62P expression in cancer patients is significantly higher than that in healthy volunteers (Fig. 1).

### Cancer cell supernatant induces platelet activation

**Fig. 2:**
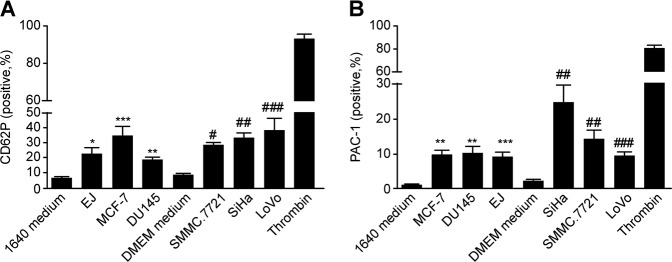
Cancer cell supernatant-induced platelet activation. Washed human platelets were preincubated with cancer cell supernatant, and then the expression of CD62P (**a**) and PAC-1 (**b**) was detected. Thrombin groups were positive control. Data are represented as the mean ± SEM for three independent experiments. *, # *P* < 0.05; **, ## *P* < 0.01; and ***, ### *P* < 0.001 compared with the appropriate RPMI 1640 or DMEM medium group

**Fig. 3:**
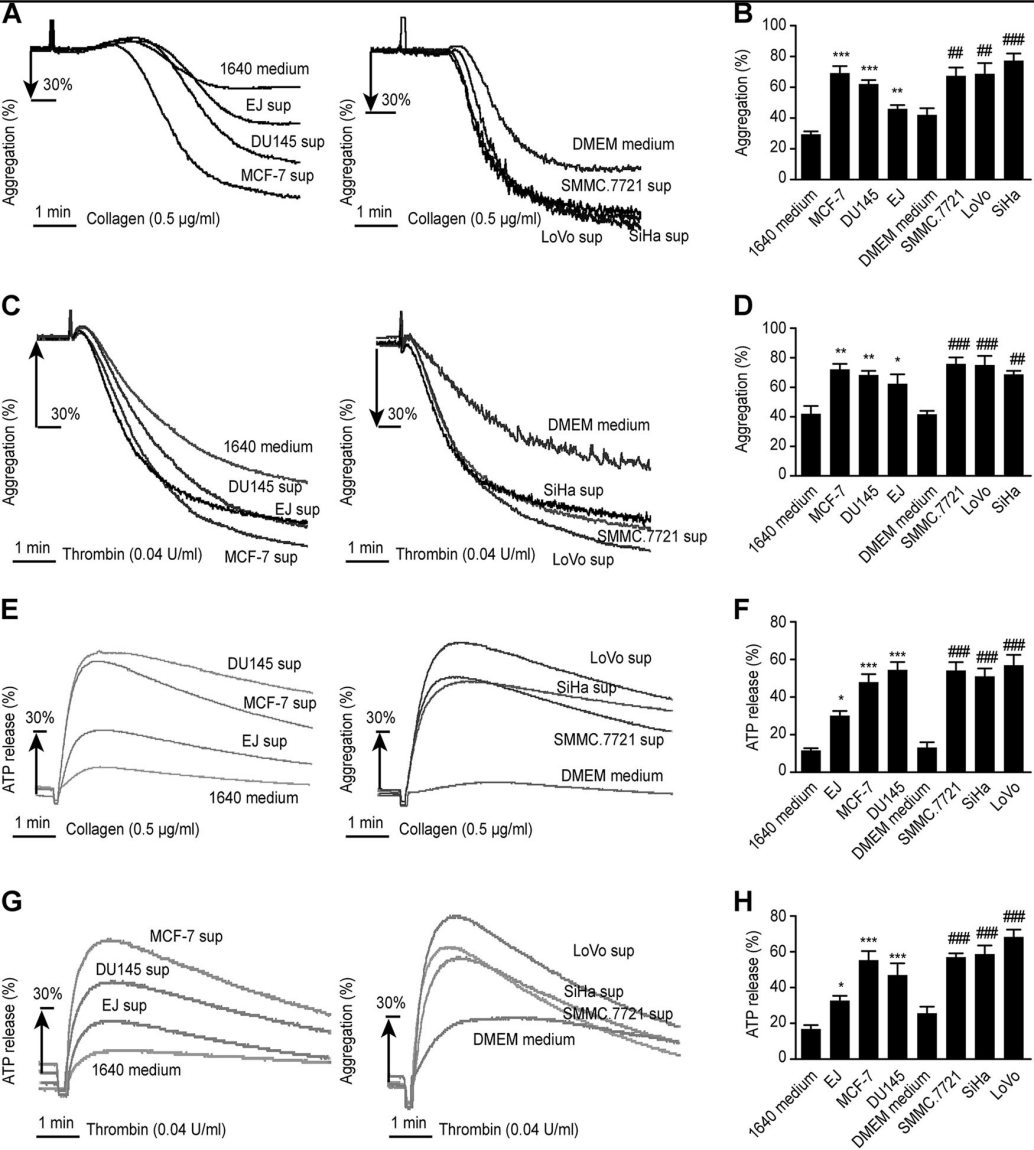
Preincubation with tumor cell supernatant promoted platelet aggregation and ATP release. Human washed platelets were pretreated with culture medium or cancer cell supernatant and CaCl_2_ (1 mM) for 15 min, and aggregation and ATP release were detected (**a**, **b**, **e**, **f**: 0.5 μg/mL collagen as agonist; **c**, **d**, **g**, **h**: 0.04 U/mL thrombin as agonist). Representative curves are shown. All experiments were repeated at least three times. Data are represented as the mean ± SEM. *, # *P* < 0.05, **, ## *P* < 0.01; and ***, ### *P* < 0.001 compared with the appropriate RPMI 1640 or DMEM medium group

We observed that cancer cell supernatant is a potent inducer of platelet activation without additional agonists. The percentage of CD62P-positive platelets in cancer cell supernatant group were much higher than that in culture medium group (Fig. 2a). We also analyzed the active form of integrin αIIbβ3, determined by binding of the monoclonal antibody PAC-1. The PAC-1-positive platelet percentage in the cancer cell supernatant group was also higher than that in the culture medium group (Fig. 2b). With an additional agonist, collagen (0.5 μg/mL) or thrombin (0.04 U/mL), the percentage of aggregated platelets in the group incubated with cancer cell supernatant was significantly higher than that in the group with culture medium incubation (Fig. 3a–d), and ATP release followed the same pattern (Fig. 3e, h). Collectively, these data indicated that platelet activation could be induced by the cancer cell supernatant, although cancer cell supernatant could not induce the aggregation of platelets directly (Supplementary Figure 1).

### Cancer cell supernatant activates platelets via FcγRIIa

**Fig. 4:**
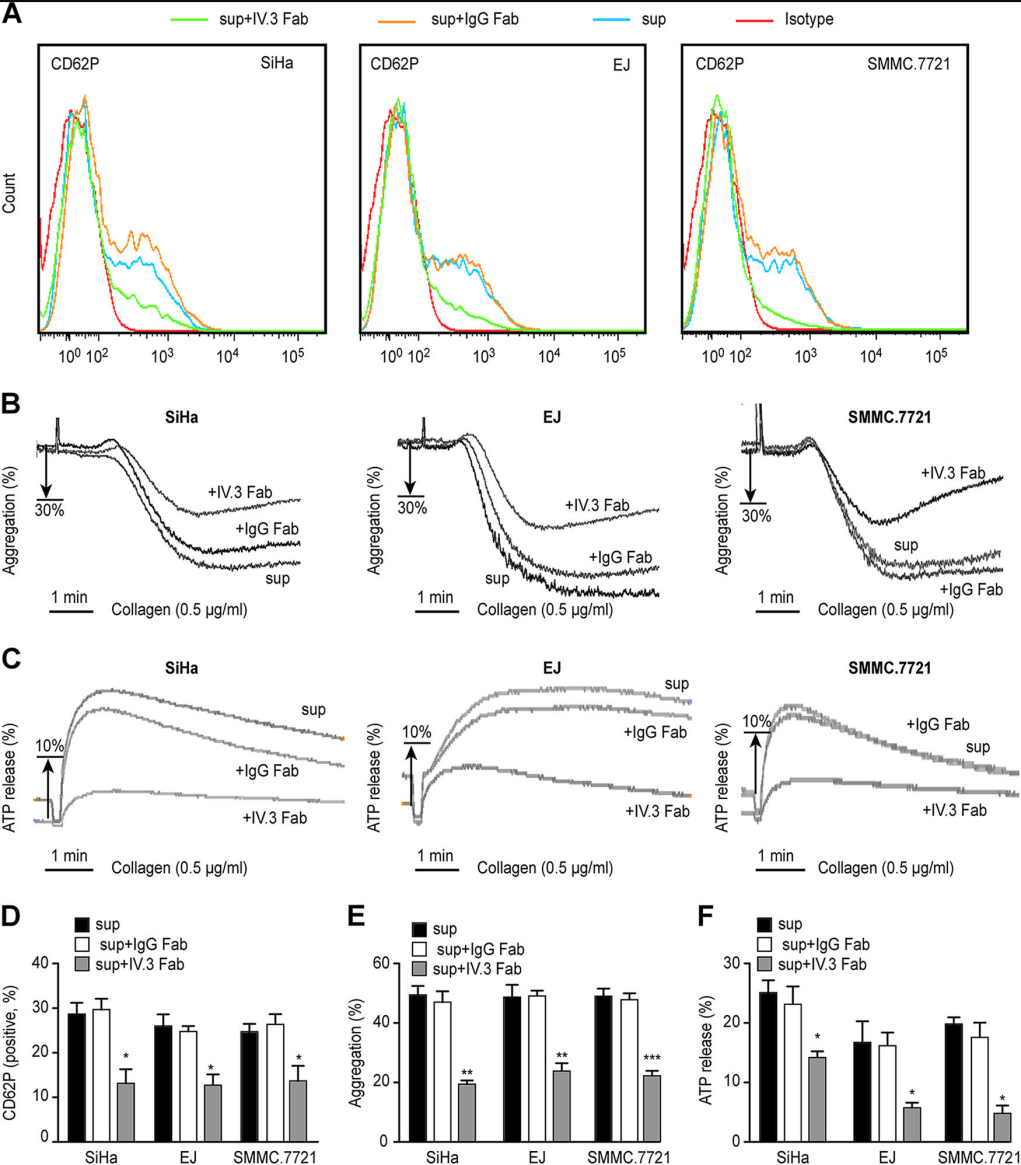
FcγRIIa was involved in platelet activation induced by cancer cell supernatant. Washed human platelets were pretreated with IV.3 Fab or IgG Fab (10 μg/mL) for 10 min and coincubated with cancer cell supernatant and CaCl_2_ (1 mM) for 15 min. The CD62P-positive platelet percentages are shown in **a**, **d**, aggregation and ATP release are shown in **b**, **e** and **c**, **f**. 0.5 μg/mL collagen was used as agonist (**b**, **c**, **e**, **f**). Data are represented as the mean ± SEM for three independent experiments. *P < 0.05; **P < 0.01; and ***P < 0.001 compared with the IgG Fab group

It has been reported that there is a direct correlation between prethrombotic state and the grade malignancy of cervical cancer^30,31^.We also found platelet activity in patients with cervical cancer was significantly higher than that in normal human platelets. To analyze the mechanism of platelet activation in the presence of cancer cell supernatant, we used several antagonists, ML-161(block PAR1), Eptifibatide (selectively blocks the platelet α II bβ3), and IV.3 Fab. IV.3 is a murine IgG2b monoclonal antibody whose Fab terminus specifically binds to the 132–137 (FSHLDP) amino acid in the second Ig-like domain of human platelet FcγRIIa, and this domain is located within the region where IgG binds^32^. When used in monomeric form, IV.3 can block the binding of the ligand to FcyRIIa. Based on this, in basic research or clinical diagnosis, IV.3 monomer or its Fab segment or F(ab′)2 is often used to study the function of FcyRIIa^33^. Here, we used IV.3 Fab as an inhibitor. We found ML-161, Eptifibatide, and IV.3 Fab all reduced CD62P expression (Supplementary Figure 2). The blocking effect of ML-161 probably by blocking the effects of thrombin, which can also be released by cancer cells^34,35^. It has been reported that αIIbβ3 was essentital for FcγRIIa signaling^36^, here we found inhibiting FcγRIIa or αIIbβ3 both reduced the expression of CD62P, we hypothesize that there may be some factors in the cancer cell culture supernatant that can affect platelet activity by acting on FcγRIIa. To further confirm the role of FcγRIIa, we used three tumor cell lines to analyze platelet CD62P expression, aggregation, and ATP release. As shown in Fig. 4a, d, coincubation with cancer cell supernatant and IgG Fab (as a control group) had no effect on CD62P expression compared to expression after supernatant treatment alone, while IV.3 Fab coincubation reduced CD62P expression significantly not only in the SiHa sup-treated group but also in EJ and SMMC.7721 sup-treated groups. The aggregation percentage (Fig. 4b, e) in the IV.3 Fab co-treated groups was also significantly reduced compared to that in the IgG Fab co-treated groups, as well as ATP release (Fig. 4c, f).

### The IgG secreted by cancer cells is mainly IgG1

**Fig. 5:**
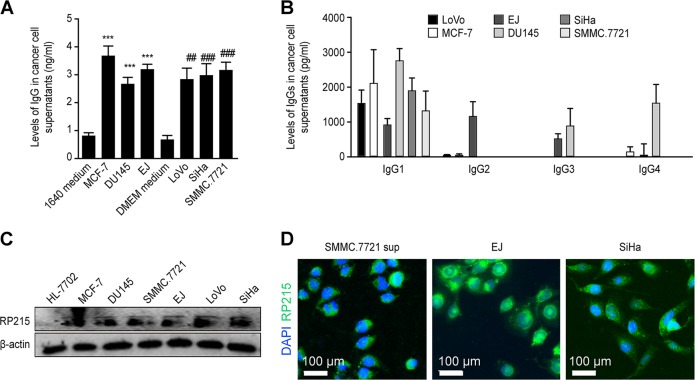
IgG secreted by tumor cells. IgG was analyzed by ELISA (**a**) and human Ig isotype array (**b**). Data are represented as the mean ± SEM for three independent experiments. ##*P* < 0.01 and ***, ###*P* < 0.001 compared with the culture medium group. **c** Expression of cancer cell-derived IgG was analyzed by RP215 antibody. Liver cell HL-7702 was used as a negative control. **d** Cancer cell-derived IgG expression in SMMC.7721, EJ and SiHa cells were analyzed by immunofluorescence, and five randomly selected fields were examined. Scale bar: 100 μm

It has been recognized that cancer cells can express IgG and that IgG has membrane-bound and secretory forms^37^. Here, we detected the IgG content in the cancer cell culture supernatants. Different cancer cells had different expression capabilities, but the levels ranged from 2 to 4 ng/mL (Fig. 5a). The IgG subtypes secreted by tumor cells were IgG1-4, but the main subtype was IgG1 (Fig. 5b). This result was consistent with the Gregory Lee report^38^ on mRNA levels. RP215 is an antibody that specifically recognizes carbohydrate-associated epitope(s) localized in the variable region of the cancer cell-expressed immunoglobulin heavy chains. It does not react with normal human IgG^39^. Using RP215, we also detected the expression of IgG in cancer cells by western blot and Immunofluorescence (Fig. 5c, d).

### Cancer cell-derived IgG mediates platelet activation

**Fig. 6:**
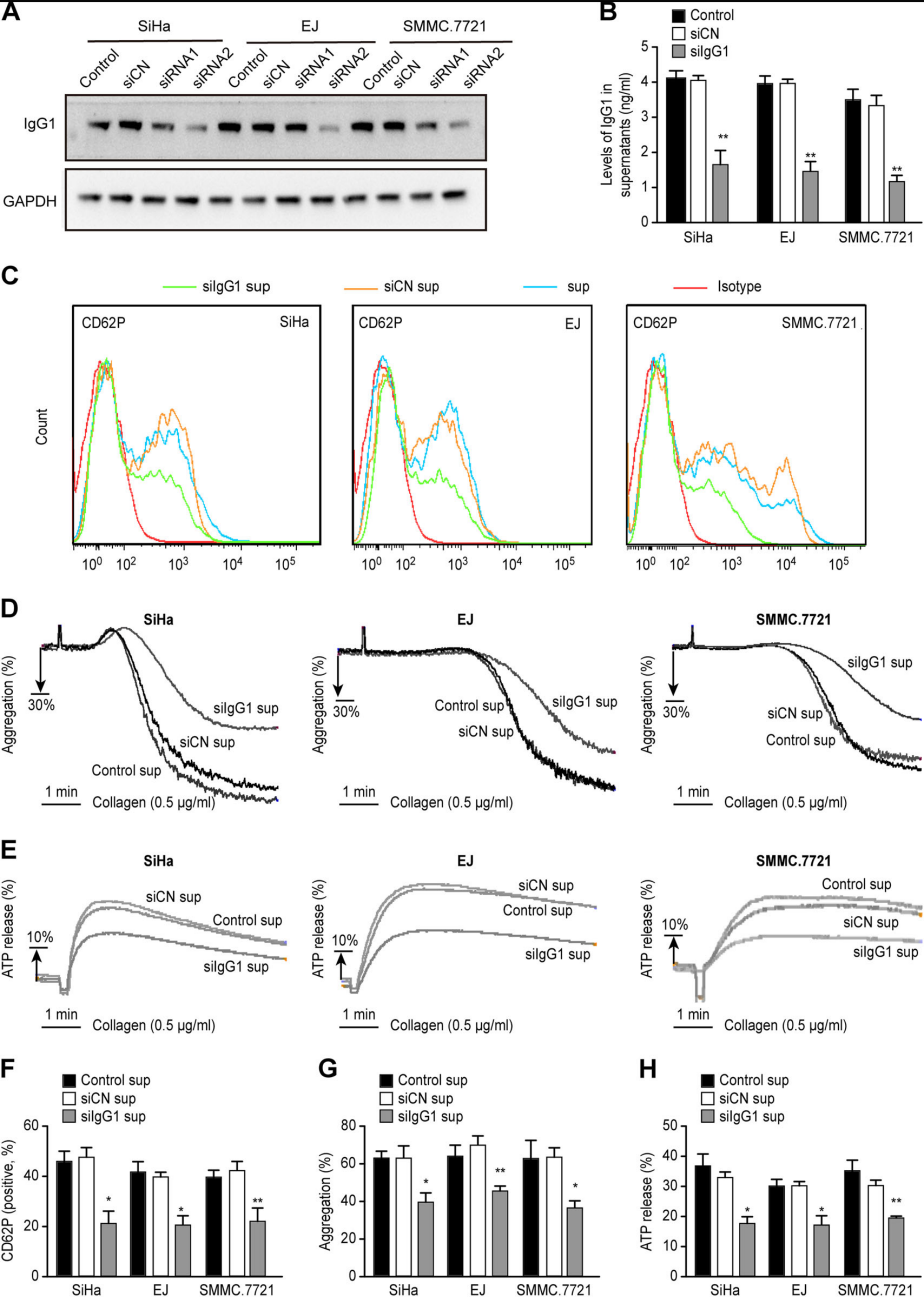
Cancer cell-derived IgG mediated platelet activation. SiHa, EJ, and SMMC.7721 cells were transfected with either control siRNA (siCN) or IgG1 siRNA for 72 h, and IgG1 expression was detected by Western blot (shown in **a**). IgG1 levels in the supernatant from siCN- treated or siIgG1-treated cells were determined by ELISA (shown in **b**). Representative traces of CD62P expression, aggregation, and ATP release triggered by siCN and siIgG1 supernatants are shown in **c**, **d**, and **e**, respectively. The corresponding histograms are shown in **f**, **g**, and **h**, respectively. 0.5 μg/mL collagen was used as agonist (**d**, **e**, **g**, **h**). *P < 0.05; **P < 0.01 compared with the siCN group. All experiments were repeated three times

Studies have shown that tumor-derived IgG can promote tumor cell proliferation and invasion^39^. However, the function of tumor-derived IgG in other aspects has not yet been clarified. We reduced the expression of IgG1 in cancer cells by siRNA treatment and detected the activation of platelets by cell supernatants. Western blotting confirmed that we efficiently knocked down IgG1 by siRNA2, but not siRNA1 (Fig. 6a) and in the following experiments we used siRNA2 to knock down IgG1 (siIgG1). ELISA analysis also confirmed IgG1 was efficiently knocked down by siIgG1 (Fig. 6b). Treatment with siIgG1 supernatants reduced CD62P expression (Fig. 6c, f). Although the platelets were stimulated by the same dose of collagen, the percentage of aggregated platelets in the group treated with siIgG1 supernatants was lower than that of the group treated with siCN supernatants (Fig. 6d, g), and ATP release followed the same pattern (Fig. 6e, h). We found that human recombinant IgG protein did not induce CD62P expression (Supplementary Figure 3). This result implied that cancer cell-derived IgG is different from normal IgG, especially in regard to platelet activation.

### Cancer cell-derived IgG1 can activate platelet FcγRIIa signaling

Blocking FcγRIIa attenuated CD62P expression induced by cancer cell supernatant, we further analyzed the effect of cancer cell supernatant on downstream signal of FcγRIIa.

**Fig. 7:**
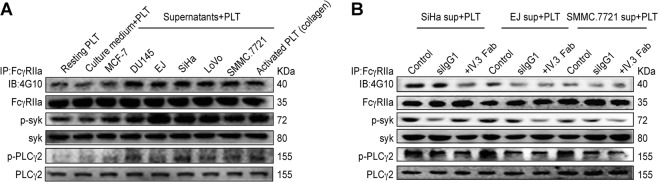
The FcγRIIa downstream signaling pathway was activated by tumor cell supernatant. Washed human platelets were incubated with culture medium or cancer cell supernatant and CaCl_2_ for 15 min and subsequently lysed. The resting platelets and collagen (0.5 μg/mL)-stimulated platelets were used as negative and positive controls, respectively. The levels of phosphorylation of FcγRIIa, syk, and PLCγ2 were analyzed, and representative results are shown in **a**. The effects of IV.3 Fab or siIgG1 on FcγRIIa and downstream signaling are shown in **b**. The experiment was repeated three times

We found cancer cell supernatant incubation increased the phosphorylation of FcγRIIa, PLCγ, and syk in platelets compared with the culture medium treatment (Fig. 7a) and pretreating platelets with IV.3 Fab reduced the phosphorylation of FcγRIIa, PLCγ, and syk (Fig. 7b), indicating that FcγRIIa-signaling pathway was involved in the activation of platelet induced by the cancer cell supernatant. The phosphorylation levels of FcγRIIa, PLCγ, and syk were also reduced when platelets were incubated with siIgG1 supernatants (Fig. 7b). These results demonstrate that cancer cell-derived IgG is involved in platelet FcγRIIa phosphorylation and signal transduction.

### Cancer cell-derived IgG interacts with platelet FcγRIIa

**Fig. 8:**
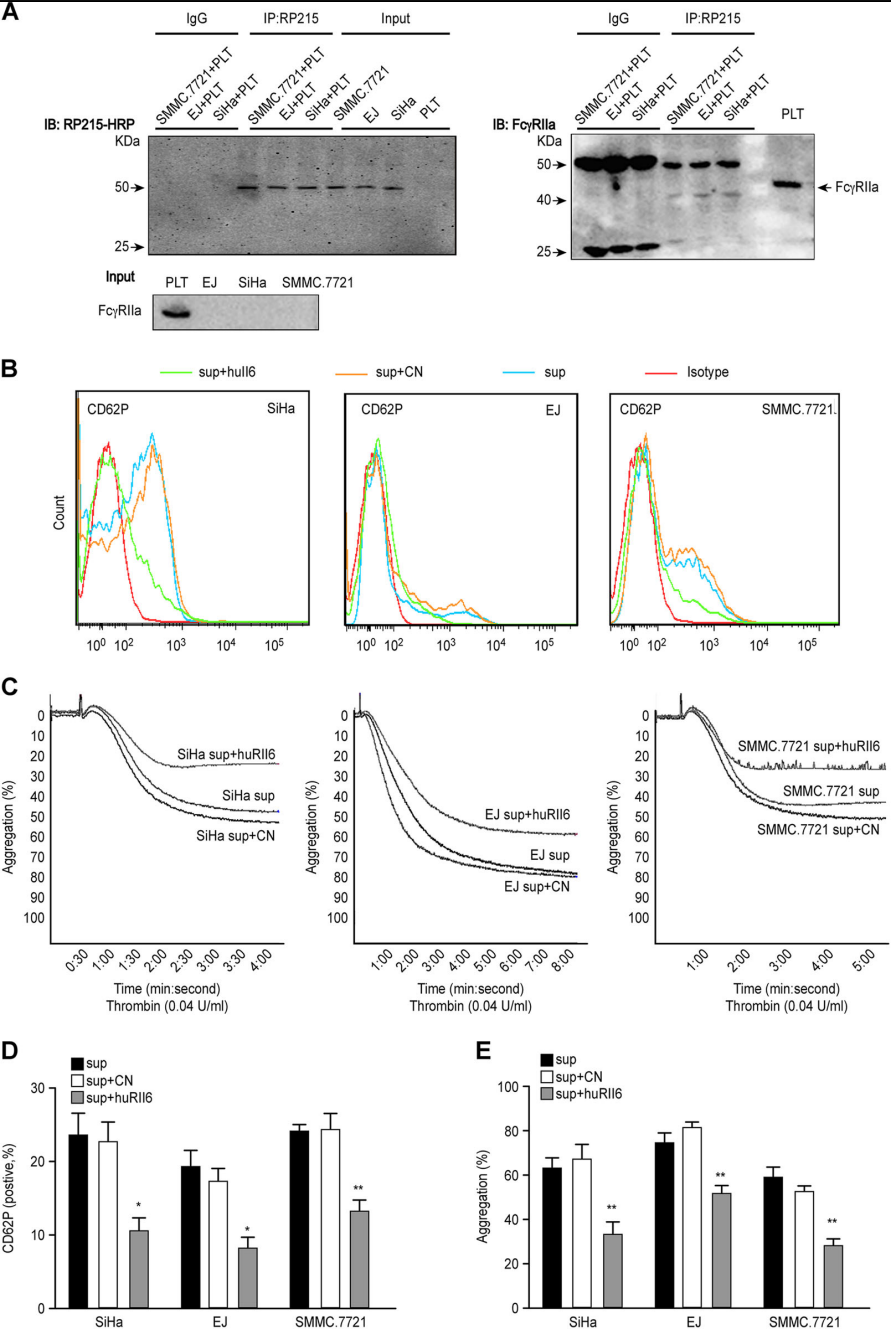
FcγRIIa binds with cancer cell-derived IgG. Coimmunoprecipitation (co-IP) of cancer cell-derived IgG with platelet FcγRIIa. **a**. Immunoprecipitated IgG and the level of IgG in cancer cells and platelets were detected by RP215-HRP antibody in the upper panel on the left, and the lower panel shows the FcγRIIa content in cancer cells and platelet lysates. The right panel shows that FcγRIIa binds to cancer cell-derived IgG after immunoprecipitating IgG with RP215. CD62P expression and the aggregation percentage of platelets treated with huRII6 or control peptide (50 μM) are shown in **b**, **d** and **c**, **e**. Thrombin (0.04 U/mL) was used as an agonist. All experiments were repeated for three times

B lymphocytes-derived IgG plays an immune role by binding to its receptor, FcγRIIa, and whether cancer cell-derived IgG can also bind to FcγRIIa is not known. Platelets were incubated with cancer cell lysates, and the mixture was immunoprecipitated with RP215. Western blot results showed that a band appeared at ~40 kDa, which was consistent with the molecular size of FcγRIIa (Fig. 8a). Because there was no cancer cell-derived IgG in platelets and RP215 cannot bind to B lymphocytes-derived IgG, we confirmed that FcγRIIa interacted with cancer cell-derived IgG. Furthermore, treatment with a small molecule inhibitor, huRII6, that was shown to be a potent competitive inhibitor of IgG binding to recombinant FcγRIIa^40^ reduced the expression of CD62P compared to the expression level after control peptide treatment (Fig. 8b, d). In addition, the aggregation of platelets was reduced by 30% in the huRII6-treated group compared with that in the control peptide-treated group (Fig. 8c, e). These data illustrated that cancer cell-derived IgG can bind to FcγRIIa and that this interaction can be blocked by huRII6.

### FcγRIIa is highly expressed by HCC platelets and activates downstream signaling

**Fig. 9:**
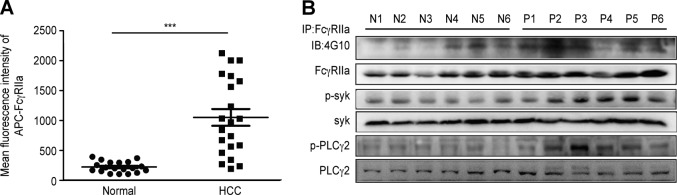
FcγRIIa was highly expressed by HCC platelets, and its downstream signaling pathway was activated. Mean fluorescence intensity of APC-FcγRIIa was analyzed by flow cytometry, and the histograms are shown (**a**). Data are represented as the mean ± SEM. ***P < 0.001 compared with normal platelets. **b** Expression and phosphorylation of FcγRIIa, syk, PLCγ2 were tested by Western blotting (N normal platelets, P patient platelets)

Flow cytometry results showed that the mean fluorescence intensity of FcγRIIa on patients’ platelets was higher than that on healthy volunteers’ platelets (1052 ± 140.7, *n* = 21 vs. 221.4 ± 20.91, *n* = 19) (Fig. 9a). In addition, phosphorylation of FcγRIIa downstream signaling molecules in patients was also stronger than that in healthy volunteers (Fig. 9b). We speculated that cancer cell-derived IgG promotes the phosphorylation of FcγRIIa and its downstream signaling molecules.

## Discussion

In the present study, we found that cancer cell culture supernatants-activated platelets and that platelets incubated with cancer cell culture supernatants were more prone to the aggregation and release of dense granules. Herein, we provide evidence that cancer cell-derived IgG is involved in the process of platelet activation by interacting directly with FcγRIIa. The mechanisms of platelet activation described here can explain the cause of abnormal platelet activity in cancer patients and provide new ideas for the prevention and treatment of tumor-associated thrombosis.

Le et al.^41^ reported that tumor cell-released HMGB1 is the key factor that interacts with TLR4 on platelets and mediates platelet–tumor cell interactions that promote metastasis. Here, we found that several cancer cell culture supernatants directly activated platelets by inducing the expression of CD62P and that FcγRIIa was involved in this process. In platelets, FcγRIIa has been identified as a transmembrane receptor responsible for mediating “outside-in” signaling through aIIbβ3^42^. Inhibitors of aIIbβ3 had a parallel profile of inhibition of tumor cell-induced platelet activation^16,43^. Similarly, blocking aIIbβ3 or inhibiting FcγRIIa also reduced the platelet activation triggered by cancer cell culture supernatants (Supplementary Figure 2). A collaborative role for the aIIbβ3 and FcγRIIa receptors may exist in the recognition of cancer cells or some other factors in supernatants.

It has been identified that platelet FcγRIIa can interact with immune complexes, specific strains of bacteria^44^, and innate pentraxins, such as the acute phase proteins serum amyloid P component and C-reactive protein^18^. Here, we showed that blocking FcγRIIa decreased the activation of platelets triggered by supernatant treatment. We hypothesize that there may be some factors in the tumor cell culture supernatant that can activate platelets via FcγRIIa. We detected no C-reactive protein in the cancer cell culture supernatants by ELISA (Supplementary Figure 4).

In recent decades, studies have shown that human tumor cells can also express IgG^19^, which has quite different functions from B lymphocytes-derived IgG. Blockade of cancer cell-derived IgG increased apoptosis in colorectal cancer cells^45^. However, the specific mechanism by which cancer cell-derived IgG promotes tumor progression has not yet been fully elucidated, and the function of tumor-derived IgG in other aspects has not yet been clarified. We found that the CD62P expression, ATP release, and aggregation of platelets induced by cancer cell culture supernatants was significantly reduced when IgG1 expression was reduced (Fig. 6). We found that the recombinant IgG protein cannot induce CD62P expression directly (Supplementary Figure 3), which is consistent with the findings of other studies^46,47^. This result demonstrates that cancer cell-derived IgG is involved in platelet activation.

RP215 is a specific antibody for cancer-derived immunoglobulins, and it does not react with normal human IgG^39^. We used RP215 for co-IP. The results showed that FcγRIIa bound with cancer cell-derived IgG (Fig. 8). Peptide huRII6 (^154^CTGNIGYTLFSSK^166^), which corresponds to the putative F-G loop of FcγRIIa, can interact with human IgG, inhibiting the binding of human IgG to soluble huFcγRIIa^40^. We found that platelets treated with huRII6 had a lower aggregation ability than the control group (Fig. 8). Similar to B lymphocytes-derived IgG binding, the F-G loop of FcγRIIa is also essential for cancer cell-derived IgG binding to FcγRIIa.

Glycans are multifunctional, they play crucial structural roles, participate in binding events, and maintain solubility and conformation^48^. Glycosylation of IgG is essential for its recognition and activation of FcγRs^48–50^. Deglycosylation of the CH2 domains of IgG abrogated sFcγRIIb binding and resulted in the lowest thermal stability accompanied by noncooperative unfolding^48,51^. The glycosylation patterns of Cancer cell-derived IgG are quite different from those of B lymphocyte-produced IgG. O-linked glycans are not detected in normal human IgG^26^ but can be detected in cancer cell-expressed IgG. In the case of N-glycans, high-mannose structure and NeuGc were detected in cancer cell-expressed IgG but not in normal human IgG^27^. In 2016, Theodoratou et al. reported that changes in glycosylation of plasma IgG correspond with significantly increased IgG proinflammatory activity, which is associated with poorer colorectal cancer prognosis, especially in late-stage cancer^52^. These findings indicate that the abnormal glycosylation of IgG is closely related to its diverse biological functions, which may explain why cancer cell-derived IgG can induce platelet activation. However, the mechanism of interaction between tumor-derived IgG and platelet FcγRIIa still needs further study.

Gregory Lee et al.^53^ reported cancer cell-expressed immunoglobulins in the plasma of cancer patients is a meaningful and beneficial pan cancer marker for cancer monitoring. Nevertheless, cancer cell-expressed immunoglobulins in the plasma could be detected at as low as 0.02 AU/mL levels presented in that study. We found that the expression level of FcγRIIa on HCC patients’ platelets was higher than that on healthy volunteers’ platelets. The high expression level of FcγRIIa may be one of the causes of abnormal platelet activity. We speculate that cancer cell-derived IgG promotes the phosphorylation of FcγRIIa and activates platelets. It is necessary to develop more sophisticated detection methods to determine the prognoses of cancers by analyzing cancer cell-derived IgG in plasma.

## Conclusion

In summary, our data clarified another way for tumor cells to activate platelets. Our data shows that the interaction between cancer cell-derived IgG and FcγRIIa on platelets leads to platelet activation. Depletion of cancer cell-derived IgG or blockage of FcγRIIa inhibited platelet activation and may be optimal approaches to prevent and treat tumor-associated thrombosis. This will abolish the vicious loop between platelet activation and cancer development.

### Availability of data and materials

All data are fully available without restriction.

## Supplementary information

Cancer cell culture supernatant did not induce platelets aggregation directly

FcγRIIa blockade attenuated platelet activation induced by cell supernatant

Recombinant IgG protein had no effect on platelet activation

Detection of C-reactive protein (CRP) in cell supernatant by ELISA

supplemental figure legends

## References

[1] Varki A (2007). Trousseau’s syndrome: multiple definitions and multiple mechanisms. Blood.

[2] Gresele P, Malvestiti M, Momi S (2018). Anti-platelet treatments in cancer: basic and clinical research. Thromb. Res..

[3] Gay LJ, Felding-Habermann B (2011). Platelets alter tumor cell attributes to propel metastasis: programming in transit. Cancer Cell.

[4] Takagi S, Takemoto A, Takami M, Oh-Hara T, Fujita N (2014). Platelets promote osteosarcoma cell growth through activation of the platelet-derived growth factor receptor-Akt signaling axis. Cancer Sci..

[5] Schlesinger M (2018). Role of platelets and platelet receptors in cancer metastasis. J. Hematol. Oncol..

[6] Lonsdorf AS (2012). Engagement of alphaIIbbeta3 (GPIIb/IIIa) with alphanubeta3 integrin mediates interaction of melanoma cells with platelets: a connection to hematogenous metastasis. J. Biol. Chem..

[7] Lowe KL, Navarro-Nunez L, Watson SP (2012). Platelet CLEC-2 and podoplanin in cancer metastasis. Thromb. Res..

[8] Smyth SS (2009). G-protein-coupled receptors as signaling targets for antiplatelet therapy. Arterioscler. Thromb. Vasc. Biol..

[9] Qi Y (2018). Novel antibodies against GPIbalpha inhibit pulmonary metastasis by affecting vWF-GPIbalpha interaction. J. Hematol. Oncol..

[10] Ekambaram P, Lambiv W, Cazzolli R, Ashton AW, Honn KV (2011). The thromboxane synthase and receptor signaling pathway in cancer: an emerging paradigm in cancer progression and metastasis. Cancer Metastas-. Rev..

[11] Farooqi AA, Siddik ZH (2015). Platelet-derived growth factor (PDGF) signalling in cancer: rapidly emerging signalling landscape. Cell Biochem. Funct..

[12] Wiesner T, Bugl S, Mayer F, Hartmann JT, Kopp HG (2010). Differential changes in platelet VEGF, Tsp, CXCL12, and CXCL4 in patients with metastatic cancer. Clin. Exp. Metastas-..

[13] He AD (2017). Platelet releasates promote the proliferation of hepatocellular carcinoma cells by suppressing the expression of KLF6. Sci. Rep..

[14] Adesanya MA, Maraveyas A, Madden LA (2016). PO-27 - thrombin generation in pancreatic cancer and multiple myeloma with use of calibrated automated thrombography. Thromb. Res..

[15] Kim S, Kunapuli SP (2011). P2Y12 receptor in platelet activation. Platelets.

[16] Mitrugno A, Williams D, Kerrigan SW, Moran N (2014). A novel and essential role for FcgammaRIIa in cancer cell-induced platelet activation. Blood.

[17] Arman M (2014). Amplification of bacteria-induced platelet activation is triggered by FcgammaRIIA, integrin alphaIIbbeta3, and platelet factor 4. Blood.

[18] Lu J (2008). Structural recognition and functional activation of FcgammaR by innate pentraxins. Nature.

[19] Kimoto Y (1998). Expression of heavy-chain constant region of immunoglobulin and T-cell receptor gene transcripts in human non-hematopoietic tumor cell lines. Genes, Chromosomes Cancer.

[20] Qiu X (2003). Human epithelial cancers secrete immunoglobulin g with unidentified specificity to promote growth and survival of tumor cells. Cancer Res..

[21] Wang J (2013). Cancer-derived immunoglobulin G promotes tumor cell growth and proliferation through inducing production of reactive oxygen species. Cell Death Dis..

[22] Wang J, Lin D, Peng H, Shao J, Gu J (2014). Cancer-derived immunoglobulin G promotes LPS-induced proinflammatory cytokine production via binding to TLR4 in cervical cancer cells. Oncotarget.

[23] Zheng J (2009). Immunoglobulin gene transcripts have distinct VHDJH recombination characteristics in human epithelial cancer cells. J. Biol. Chem..

[24] Zhu X (2010). Distinct regulatory mechanism of immunoglobulin gene transcription in epithelial cancer cells. Cell. Mol. Immunol..

[25] Lee G, Cheung AP, Li B, Ge B, Chow PM (2012). Molecular and immuno-characteristics of immunoglobulin-like glycoproteins in cancer cell-expressed biomarker, CA215. Immunol. Investig..

[26] Arnold JN, Wormald MR, Sim RB, Rudd PM, Dwek RA (2007). The impact of glycosylation on the biological function and structure of human immunoglobulins. Annu. Rev. Immunol..

[27] Lee G, Azadi P (2012). Peptide mapping and glycoanalysis of cancer cell-expressed glycoproteins CA215 recognized by RP215 monoclonal antibody. J. Carbohyd. Chem..

[28] Liu G (2016). Antiplatelet activity of chrysin via inhibiting platelet alphaIIbbeta3-mediated signaling pathway. Mol. Nutr. Food Res..

[29] Liang ML (2015). Pentamethylquercetin (PMQ) reduces thrombus formation by inhibiting platelet function. Sci. Rep..

[30] Sun YH (2015). Analysis of relationships between prethrombotic states and cervical cancer. Asian Pac. J. Cancer Prev..

[31] Satoh T (2013). Incidence of venous thromboembolism before treatment in cervical cancer and the impact of management on venous thromboembolism after commencement of treatment. Thromb. Res..

[32] Tridandapani S (2002). Regulated expression and inhibitory function of Fcgamma RIIb in human monocytic cells. J. Biol. Chem..

[33] Rosenfeld SI (1985). Human platelet Fc receptor for immunoglobulin G. Identification as a 40,000-molecular-weight membrane protein shared by monocytes. J. Clin. Investig..

[34] Adesanya MA, Maraveyas A, Madden L (2017). Differing mechanisms of thrombin generation in live haematological and solid cancer cells determined by calibrated automated thrombography. Blood Coagul. Fibrinolysis.: Int. J. Haemost. Thromb..

[35] Heinmoller E (1995). Tumor cell-induced platelet aggregation in vitro by human pancreatic cancer cell lines. Scand. J. Gastroenterol..

[36] Boylan B (2008). Identification of FcgammaRIIa as the ITAM-bearing receptor mediating alphaIIbbeta3 outside-in integrin signaling in human platelets. Blood.

[37] Lee G, Zhu MG, Ge BX, Potzold S (2012). Widespread expressions of immunoglobulin superfamily proteins in cancer cells. Cancer Immunol. Immun..

[38] Lee G, Ge BX (2009). Cancer cell expressions of immunoglobulin heavy chains with unique carbohydrate-associated biomarker. Cancer Biomark..

[39] Liao Q (2015). Aberrant high expression of immunoglobulin G in epithelial stem/progenitor-like cells contributes to tumor initiation and metastasis. Oncotarget.

[40] Xi J (2012). Increased survival and reduced renal injury in MRL/lpr mice treated with a human Fcgamma receptor II (CD32) peptide. Immunology.

[41] Yu LX (2014). Platelets promote tumour metastasis via interaction between TLR4 and tumour cell-released high-mobility group box1 protein. Nat. Commun..

[42] Zhi H (2013). Cooperative integrin/ITAM signaling in platelets enhances thrombus formation in vitro and in vivo. Blood.

[43] Steinert BW, Tang DG, Grossi IM, Umbarger LA, Honn KV (1993). Studies on the role of platelet eicosanoid metabolism and integrin alpha IIb beta 3 in tumor-cell-induced platelet aggregation. Int. J. Cancer.

[44] Cox D, Kerrigan SW, Watson SP (2011). Platelets and the innate immune system: mechanisms of bacterial-induced platelet activation. J. Thromb. Haemost..

[45] Niu N (2012). IgG expression in human colorectal cancer and its relationship to cancer cell behaviors. PloS One.

[46] Boilard E (2014). Influenza virus H1N1 activates platelets through FcgammaRIIA signaling and thrombin generation. Blood.

[47] Kang J, Cabral C, Kushner L, Salzman EW (1993). Membrane glycoproteins and platelet cytoskeleton in immune complex-induced platelet activation. Blood.

[48] Mimura Y (2001). Role of oligosaccharide residues of IgG1-Fc in Fc gamma RIIb binding. J. Biol. Chem..

[49] Mimura Y (2000). The influence of glycosylation on the thermal stability and effector function expression of human IgG1-Fc: properties of a series of truncated glycoforms. Mol. Immunol..

[50] Krapp S, Mimura Y, Jefferis R, Huber R, Sondermann P (2003). Structural analysis of human IgG-Fc glycoforms reveals a correlation between glycosylation and structural integrity. J. Mol. Biol..

[51] Tao MH, Morrison SL (1989). Studies of aglycosylated chimeric mouse-human IgG. Role of carbohydrate in the structure and effector functions mediated by the human IgG constant region. J. Immunol..

[52] Theodoratou E (2016). Glycosylation of plasma IgG in colorectal cancer prognosis. Sci. Rep..

[53] Lee G (2010). Positive identification of CA215 pan cancer biomarker from serum specimens of cancer patients. Cancer Biomark..

