# Supplementary figures and images for: Cancer cell-derived immunoglobulin G activates platelets by binding to platelet FcγRIIa
Source: Cell Death Dis. 2019 Jan 28;10(2):87. doi: 10.1038/s41419-019-1367-x (PMC6349849; doi:10.1038/s41419-019-1367-x)

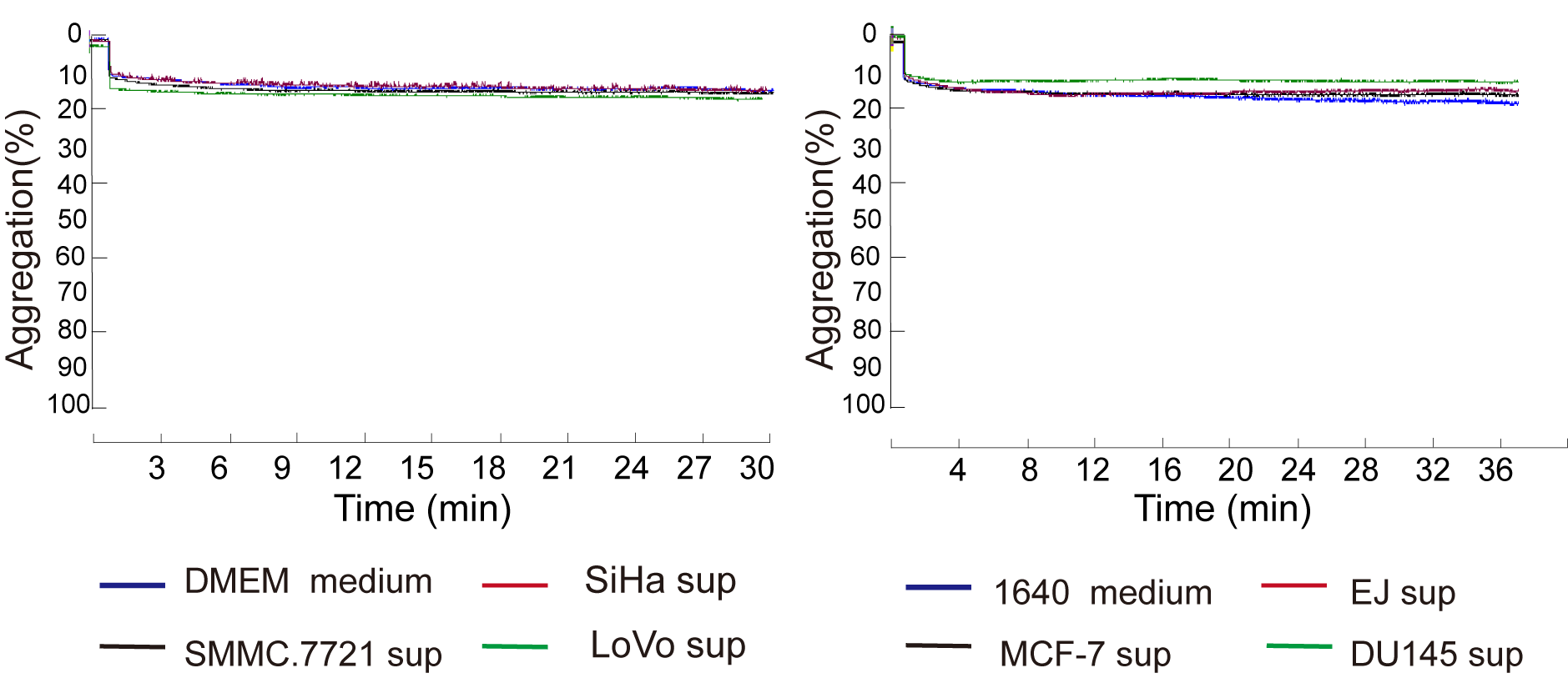

Supplement: Supplementary file 1 — Cancer cell culture supernatant did not induce platelets aggregation directly [file 41419_2019_1367_MOESM1_ESM.tif]

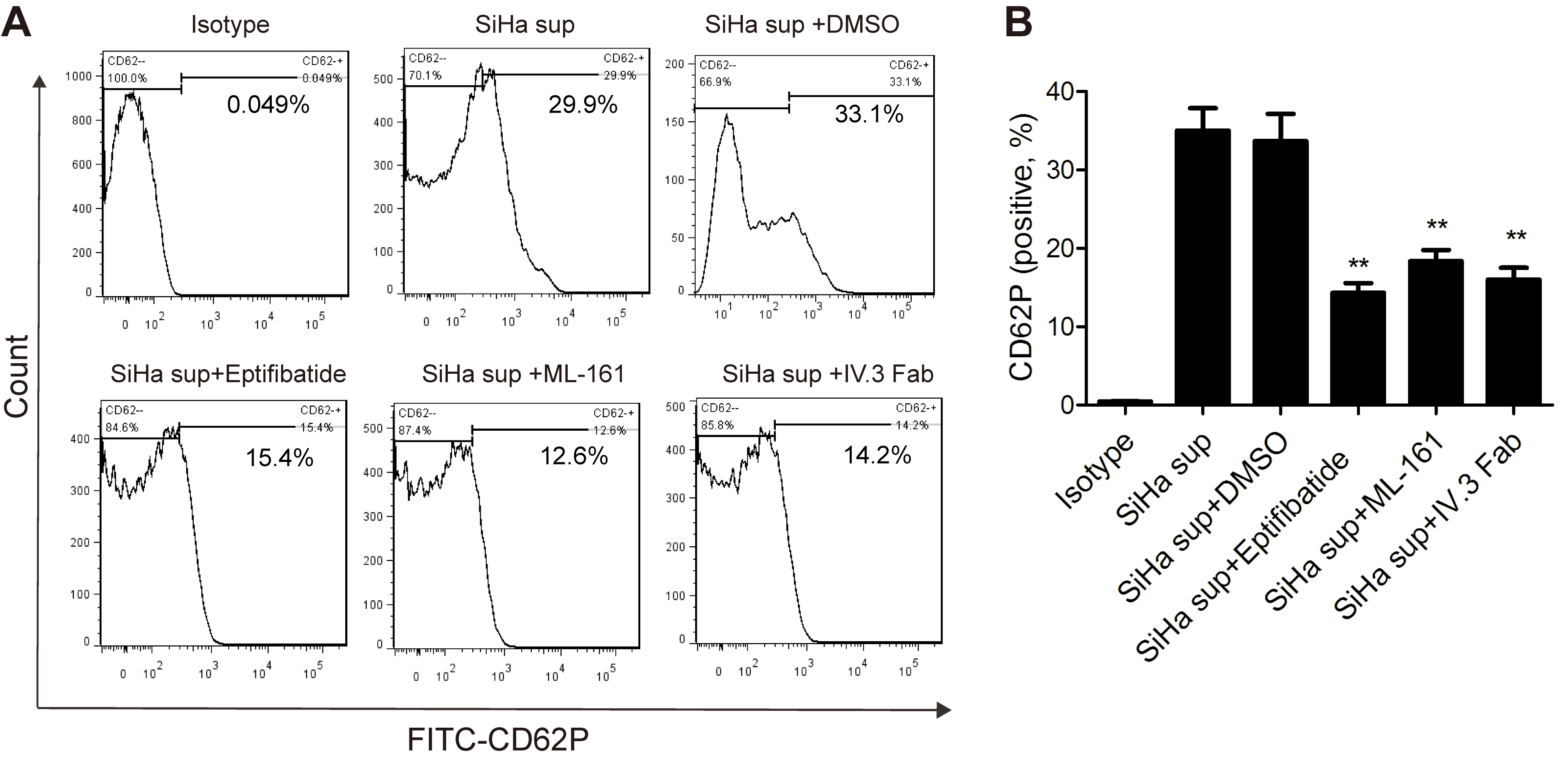

Supplement: Supplementary file 2 — FcγRIIa blockade attenuated platelet activation induced by cell supernatant [file 41419_2019_1367_MOESM2_ESM.tif]

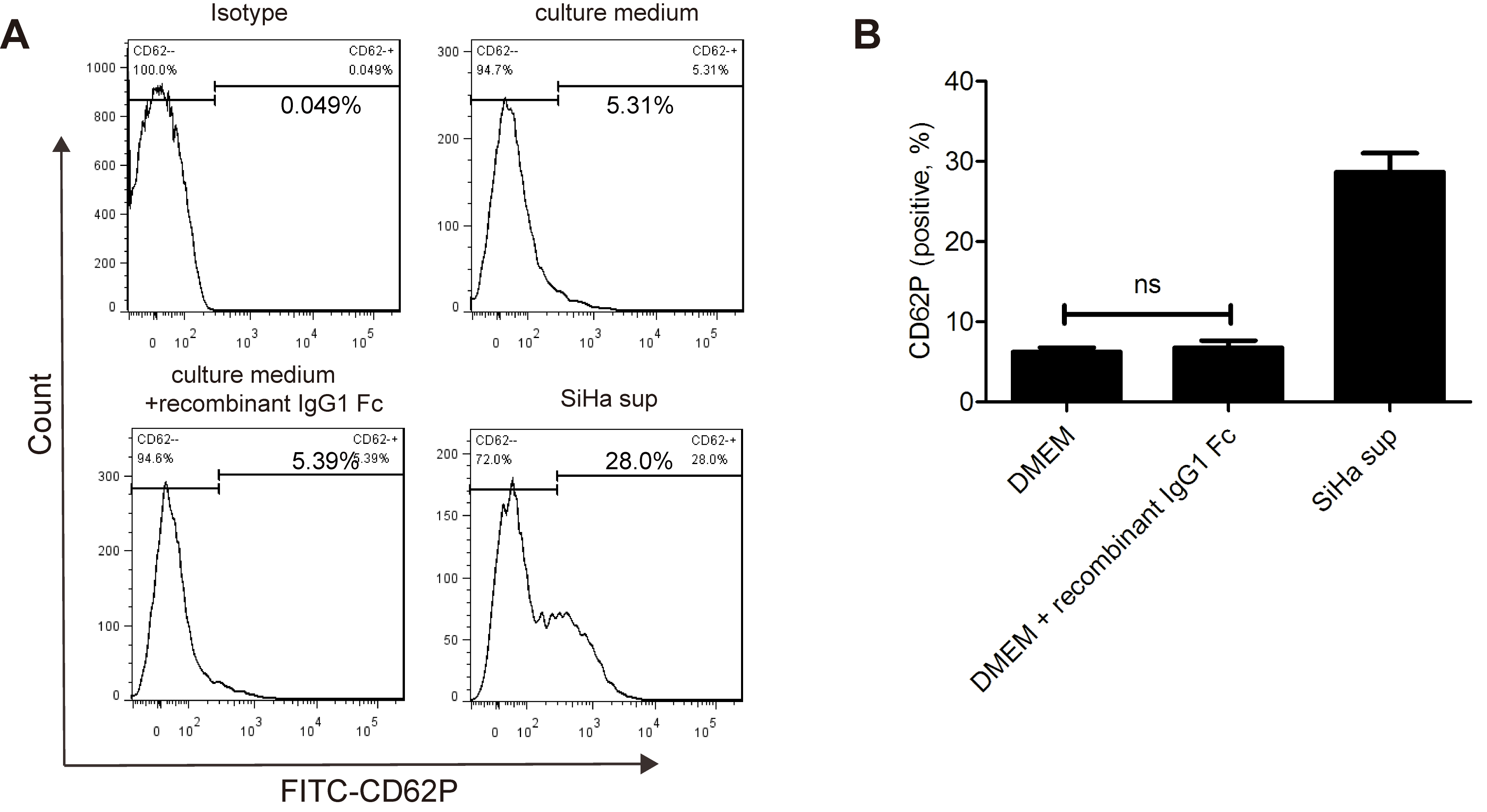

Supplement: Supplementary file 3 — Recombinant IgG protein had no effect on platelet activation [file 41419_2019_1367_MOESM3_ESM.tif]

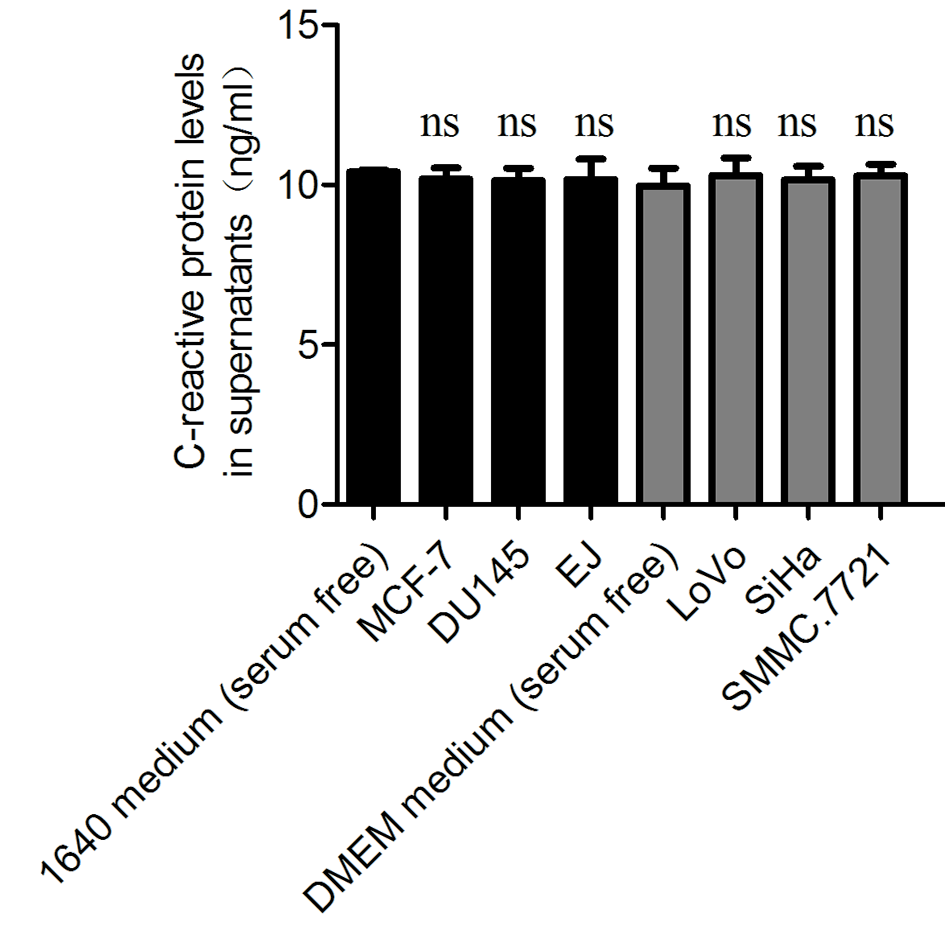

Supplement: Supplementary file 4 — Detection of C-reactive protein (CRP) in cell supernatant by ELISA [file 41419_2019_1367_MOESM4_ESM.tif]
