# Supplementary material for: Cancer cell-derived immunoglobulin G activates platelets by binding to platelet FcγRIIa
Source: Cell Death Dis. 2019 Jan 28;10(2):87. doi: 10.1038/s41419-019-1367-x (PMC6349849; doi:10.1038/s41419-019-1367-x)
Supplement: Supplementary file 5 — supplemental figure legends [file 41419_2019_1367_MOESM5_ESM.docx]

**Supplementary Figure1. Cancer cell culture supernatant did not induce platelets aggregation directly.**

Human washed platelets (450×10^9^/L, 200μl) were pre-incubated with 1mM CaCl_2_ for 5min, 100μl cancer cell culture supernatant was added to platelet suspension after the adjustment of the baseline. The aggregation percentage was recorded and the representative tracing were shown.

**Supplementary Figure2. FcγRIIa blockade attenuated platelet activation induced by cell supernatant.**

Human washed platelets were pre-incubated with ML-161(block PAR1, 10μg/ml), Eptifibatide (inhibit αIIbβ3, 5μg/ml), IV.3 Fab (10μg/ml) for 15min before supernatant incubation. The representative tracing and histogram of CD62P expression were shown in A and B. Dates are represented as Mean ± SEM for three independent experiments. **, *P* < 0.01 compared with the SiHa supernatant group.

**Supplementary Figure3. Recombinant IgG protein had no effect on platelet activation.**

Human washed platelets were pre-incubated with culture medium, culture medium+ recombinant IgG1 Fc (10μg/ml) or SiHa supernatant for 15 min and detected the expression of CD62P. The representative tracing were shown. All experiments were repeated at least three times. ns: no significant difference.

**Supplementary Figure4.** **Detection of C-reactive protein (CRP) in cell supernatant by ELISA.**

Cancer cell culture supernatants were collected and analyzed the CRP by ELISA. The levels of CRP in tumor cell supernatants group were compared with the culture medium group. The experiment was repeated three times. ns: no significant difference.
